# Novel class of peptides disintegrating biological membranes to aid in the characterization of membrane proteins

**DOI:** 10.1016/j.jbc.2024.107154

**Published:** 2024-03-11

**Authors:** Václav Hořejší, Pavla Angelisová, Jana Pokorná, Tatsiana Charnavets, Oldřich Benada, Tomáš Čajka, Tomáš Brdička

**Affiliations:** 1 Institute of Molecular Genetics of the Czech Academy of Sciences, Prague, Czech Republic; 2 Institute of Biotechnology of the Czech Academy of Sciences, BIOCEV, Vestec, Czech Republic; 3 Institute of Microbiology of the Czech Academy of Sciences, Prague, Czech Republic; 4 Institute of Physiology of the Czech Academy of Sciences, Prague, Czech Republic

**Keywords:** peptides, lipid raft, membrane, membrane proteins, lymphocyte, leukocyte

## Abstract

Styrene-maleic acid (SMA) and similar amphiphilic copolymers are known to cut biological membranes into lipid nanoparticles/nanodiscs containing membrane proteins apparently in their relatively native membrane lipid environment. Our previous work demonstrated that membrane raft microdomains resist such disintegration by SMA. The use of SMA in studying membrane proteins is limited by its heterogeneity and the inability to prepare defined derivatives. In the present paper, we demonstrate that some amphiphilic peptides structurally mimicking SMA also similarly disintegrate cell membranes. In contrast to the previously used copolymers, the simple peptides are structurally homogeneous. We found that their membrane-disintegrating activity increases with their length (reaching optimum at 24 amino acids) and requires a basic primary structure, that is, (XXD)n, where X represents a hydrophobic amino acid (optimally phenylalanine), D aspartic acid, and n is the number of repeats of these triplets. These peptides may provide opportunities for various well-defined potentially useful modifications in the study of membrane protein biochemistry. Our present results confirm a specific character of membrane raft microdomains.

Biochemical studies of membrane receptors and other membrane proteins (adhesion molecules, ion channels, transporters, etc.) often require their solubilization in mild detergents, such as octyl glucoside, Triton X100, Brij-series, NP-40, CHAPS, etc. Following such treatment of the membranes (or whole cells), membrane proteins are present in mixed micelles, in which their hydrophobic transmembrane domains are surrounded mostly by the detergent molecules. Depending on the nature of the detergent and details of the solubilization procedure, these micelles may also contain membrane lipids.

Cell membranes are known to exhibit distinct lateral heterogeneity—lipids and proteins form dynamic “microdomains” or “nanodomains” of various sizes and stability where transmembrane domains of proteins associate with specific lipids forming lipid shells. A distinct and probably heterogeneous type of such membrane microdomains (or rather nanodomains) are the so-called membrane rafts. They selectively concentrate a specific set of functionally relevant proteins (mostly lipidated) and (glyco)lipids (reviewed in (1)). Membrane rafts exhibit selective resistance to solubilization by certain detergents, for example, Triton X100, Brij-series, NP-40, or CHAPS. This property has been exploited in several widely used methods of their isolation. However, the use of detergents may produce more or less significant artifacts due to the possible loss of loosely associated components of supramolecular complexes, especially those dependent on interactions with specific membrane lipids. Furthermore, the composition and properties of detergent-resistant membrane fragments originating from membrane rafts are also dependent on the nature of the detergent and conditions used (2).

Because of the inherent problems connected with the use of detergents for isolation and biochemical studies of membrane proteins, alternative methods for disintegrating cell membranes have been sought. A suitable alternative is the use of styrene-maleic acid (SMA) copolymers (3, 4). The amphiphilic molecules of SMA spontaneously incorporate into membranes and cut them into “nanodiscs” in which the polymer forms an annulus surrounding and stabilizing, in a presumably native state, a small area of the lipid bilayer (approx. 12 nm in diameter). Apparently, the phenyl moieties of the copolymer intercalate among the lipid molecules while the carboxylate groups interact with the aqueous environment and possibly also with the lipid polar head-groups (5, 6). These nanodiscs, also called SMA-lipid particles, are relatively stable and do not need the presence of a free soluble copolymer in the solution. Similar to detergents, the membrane raft–associated proteins are not fully solubilized by SMA or other similar copolymers and remain in larger copolymer-resistant membrane fragments (7, 8). The proteins contained in the nanodiscs thus behave essentially like soluble (lipo)proteins. A number of membrane proteins and their complexes have been successfully purified from SMA-solubilized membranes (5, 6, 9, 10, 11, 12, 13).

In addition to the originally used and commercially available SMA copolymers, multiple other similar types of copolymers, including variously derivatized SMA and other copolymers, were used for the disintegration of cell membranes (see an overview by Brown *et al*. (14)). In our recent studies, we used SMA to characterize membrane proteins present within and outside of membrane rafts in T cells and other cell types (7, 15). We also demonstrated similar membrane-disintegrating properties of other amphiphilic copolymers (8, 16).

The currently used SMA and majority of similar copolymers suffer from a problem; due to the statistical nature of the copolymerization reactions, they are more or less heterogeneous, both in size and sequence of the monomer units. Furthermore, the possibilities of simple preparation of well-defined derivatives of these copolymers are rather limited. When thinking more generally about the design of compounds structurally similar to SMA, we reasoned that we could employ specific peptides structurally mimicking SMA, namely those containing suitably alternating phenyl and carboxylic groups (e.g. composed of properly ordered phenylalanine and aspartic or glutamic acid residues). Indeed, as shown below, some of these peptides solubilize membranes as expected and, in some respects, particularly due to their perfect structural homogeneity, may represent a potentially superior alternative to SMA and similar synthetic copolymers.

## Results

### The SMA-mimicking FFD8 peptide solubilizes Jurkat cell membranes

**Figure 1:**
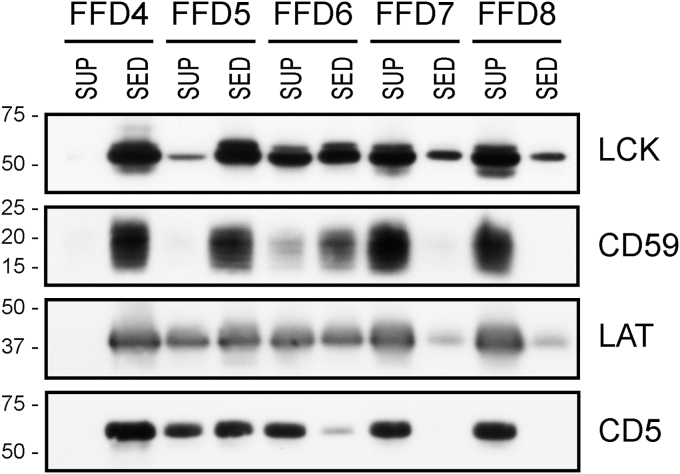
**Solubilization of Jurkat cell membranes by the FFDn peptides.** The Jurkat cell membranes were treated with the lysis buffer containing 1% (10 mg/ml) peptide, and the lysate was subjected to high-speed centrifugation. Four typical membrane proteins (LCK, CD59, LAT, CD5) were detected by immunostaining in the sediments and supernatants of the peptide-treated membrane samples. LCK, CD59, and LAT are characteristic membrane raft components, while CD5 is a non-raft protein. Only the relevant parts of the blots are shown, corresponding to the area around the MW of the respective proteins. Positions of relevant m. w. standards (in kDa) are shown at the *left*.

Some of the limitations of the SMA-like copolymers could be theoretically minimized if peptide mimetics are used instead. We hypothesized that peptides composed of repeated blocks containing various sequences of phenylalanine (F) and aspartic acid (D) may share basic chemical features with the SMA copolymers, that is, a backbone decorated with hydrophobic phenyl groups followed by carboxyls. To test this idea, we first used peptides composed of 4 to 8 blocks of amino acids, each comprising the sequence FFD (FFDFFDFFDFFD [“FFD4”] through FFDFFDFFDFFDFFDFFDFFDFFD [“FFD8”]). As shown in Figure 1, the efficiency of Jurkat T cell membrane solubilization increased with the number of the FFD repeats, FFD8 solubilizing the membrane proteins completely. The same results were obtained also with the FFD9 and FFD10 peptides (not shown).

**Figure 2:**
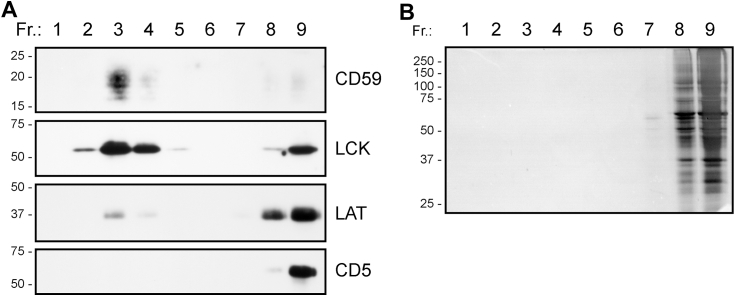
**Distribution of the indicated Jurkat cell membrane proteins in the fractions from density gradient ultracentrifugation.***A*, Jurkat cell membranes were solubilized by 1% FFD8, the lysate was fractionated by density gradient ultracentrifugation, and the indicated proteins in the fractions were detected by immunoblotting. The fractions are numbered from the *top* of the gradient. Only the relevant parts of the blots are shown, corresponding to the area around the MW of the respective proteins. *B*, the fractions from the gradient were analyzed by SDS PAGE and proteins visualized by silver staining (m.w. standards positions in kDa at the *left*). As expected, vast majority of the proteins are present in the *bottom* fractions of the gradient.

As mentioned above, in our previous studies, we used density gradient ultracentrifugation to demonstrate that the copolymer SMA efficiently solubilizes, presumably into nanodiscs, most membrane proteins except for those present in membrane raft microdomains, which remain in larger and buoyant SMA-resistant membrane fragments (7, 15). As shown in Figure 2, very similar results were obtained with Jurkat cell membranes disintegrated by 1% FFD8—the membrane raft markers (LCK and CD59) were also largely present in peptide-resistant membrane fragments (PRMs) in the top fractions of the gradient. The majority of LAT, all of the nonraft marker CD5, and the great majority of total proteins were present in the bottom fractions.

**Figure 3:**
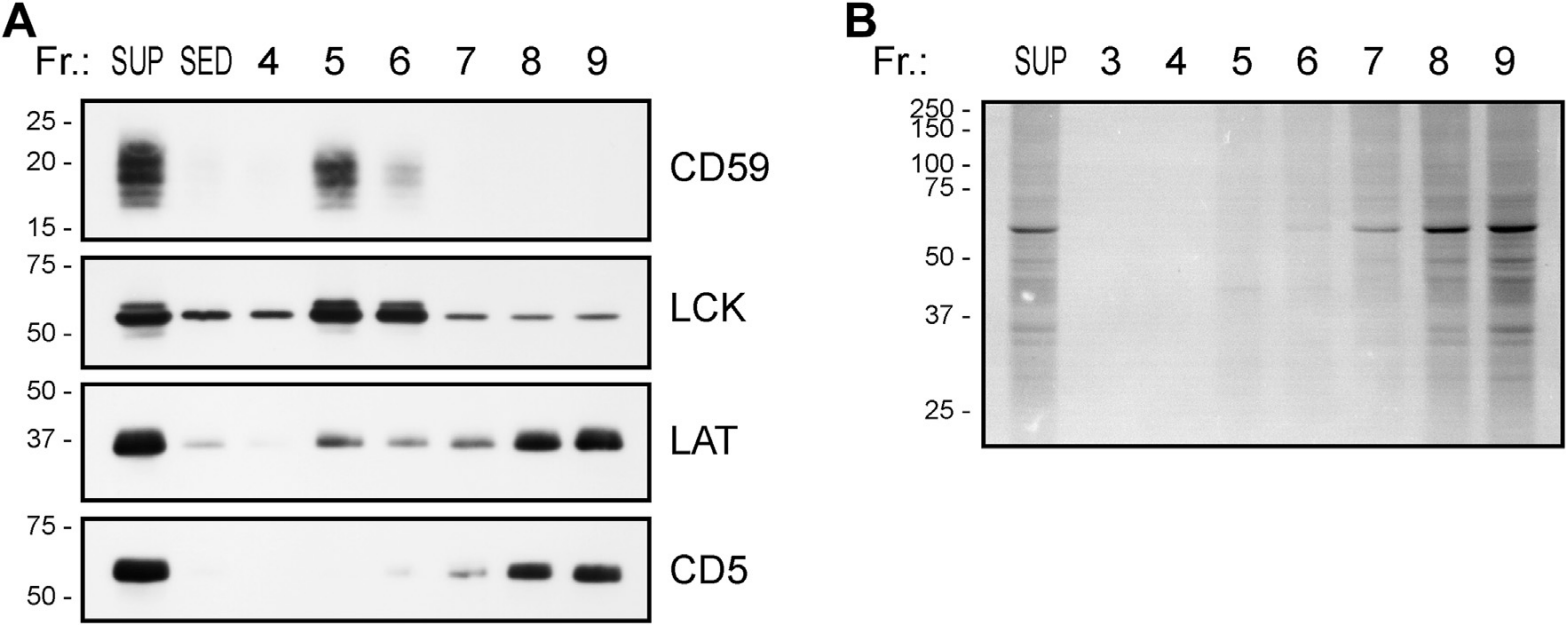
**Distribution of the indicated Jurkat cell membrane proteins in the gel filtration fractions.** Jurkat cell membranes were solubilized by 1% FFD8, the lysates were fractionated by gel filtration on Sepharose 4B, and the indicated proteins in the fractions were detected by immunoblotting (*A*). Total proteins were detected by silver staining of the gel (positions and m.w. (in kDa) of standards are shown on the *left*) (*B*). SUP, supernatant and SED sediment of the solubilized membrane sample.

Furthermore, we subjected the Jurkat cell membranes lysed by FFD8 to gel filtration on Sepharose 4B. The results shown in Figure 3 demonstrate that the vast majority of membrane proteins were present in the relatively low-molecular-weight fractions. In contrast, the raft-associated proteins were primarily detected in the fractions corresponding to much larger PRMs, similar to what has been shown previously for SMA (7).

**Figure 4:**
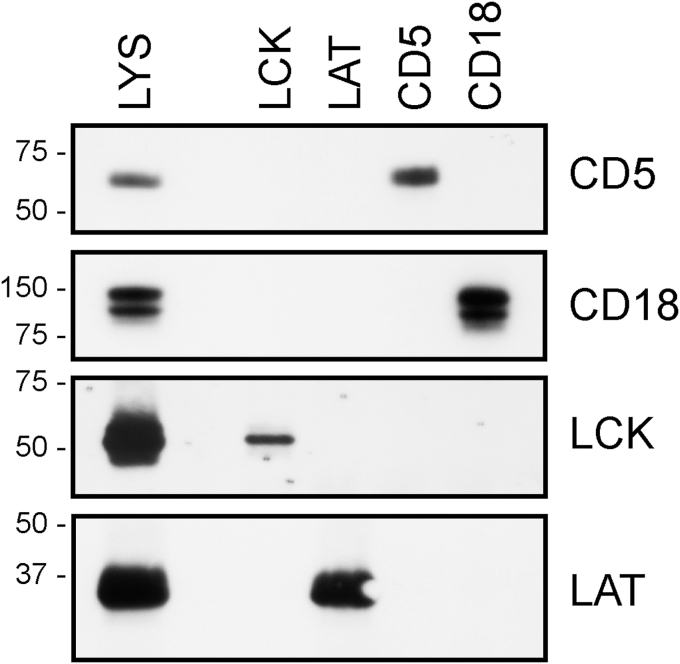
**Immunoprecipitation of membrane proteins solubilized by FFD8.** The proteins indicated at the *right*-hand side were immuno-isolated from the Jurkat cell membrane FFD8 lysate (LYS) on the bead-immobilized mAbs recognizing the membrane proteins indicated at the *top*. The immunoprecipitates were analyzed by SDS PAGE followed by Western blotting. Positions of relevant m. w. standards (in kDa) are shown at the *left*.

Next, we tested whether the membrane proteins solubilized by FFD8 can be immuno-isolated from the cell membrane lysate. As shown in Figure 4, CD5, LAT, and CD18 could be readily immuno-isolated; the yield of LCK was low, probably because this protein is mostly present in large, presumably raft-derived complexes which poorly penetrate into the Protein A/G PLUS-Sepharose beads used for the immunoisolation.

**Figure 5:**
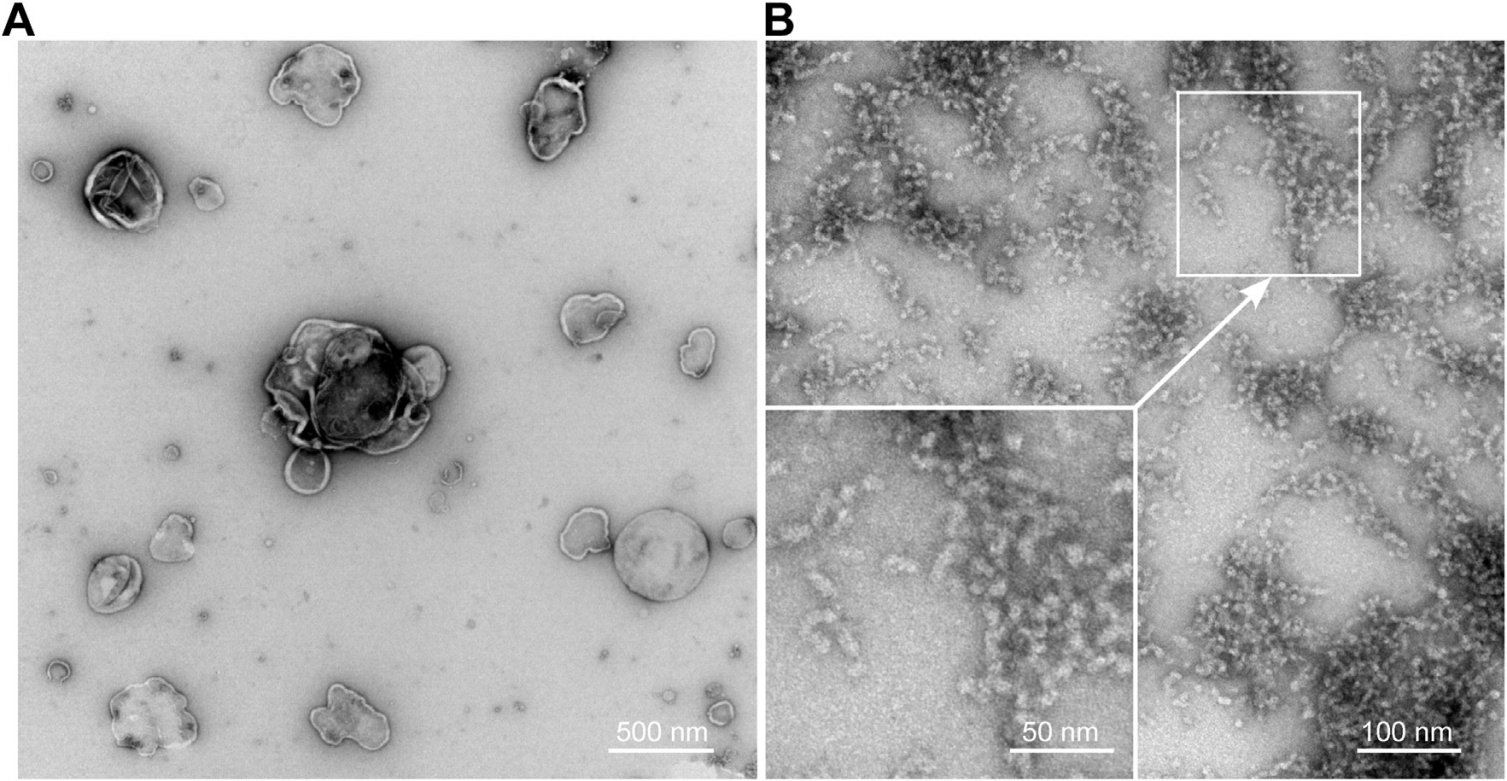
**Transmission electron microscopy.***N*egative staining of fraction 2 (*A*) and 9 (*B*), respectively, from density gradient ultracentrifugation of Jurkat cell membranes solubilized by FFD8 (the same material as examined in Fig. 2*A*).

Furthermore, we examined the fractions from the density gradient ultracentrifugation of Jurkat cell membranes solubilized by FFD8 by transmission electron microscopy (Fig. 5). As expected, the results were similar to those observed in our previous study (7) employing the SMA copolymer for the disintegration of Jurkat cell membranes. While the bottom fraction of the gradient mainly contained small objects of the shape and size of “nanodiscs,” the top fraction (accumulating some typical membrane raft proteins, cf. Fig. 2) contained much larger membrane fragments.

### Lipid composition of the solubilization-resistant membrane fragments

**Figure 6:**
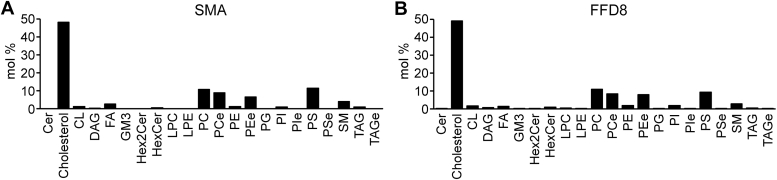
**Comparison of lipid composition of the membrane fragments resistant to SMA *versus* FFD8.** Molar percentage distribution of lipid classes in (*A*) SMA and (*B*) FFD8 membranes. Cer, ceramide; CL, cardiolipin; DAG, diacylglycerol; FA, fatty acids; GM3, ganglioside GM3; Hex2Cer, dihexosylceramide; HexCer, hexosylceramide; LPC, lysophosphatidylcholine; LPE, lysophosphatidylethanolamine; PC, phosphatidylcholine; PCe, ether-linked phosphatidylcholine; PE, phosphatidylethanolamine; PEe, ether-linked phosphatidylethanolamine; PG, phosphatidylglycerol; PI, phosphatidylinositol; PS, phosphatidylserine; PSe, ether-linked phosphatidylserine; SM, sphingomyelin; SMA, styrene-maleic acid; TAG, triacylglycerol; TAGe, ether-linked triacylglycerol.

**Figure 7:**
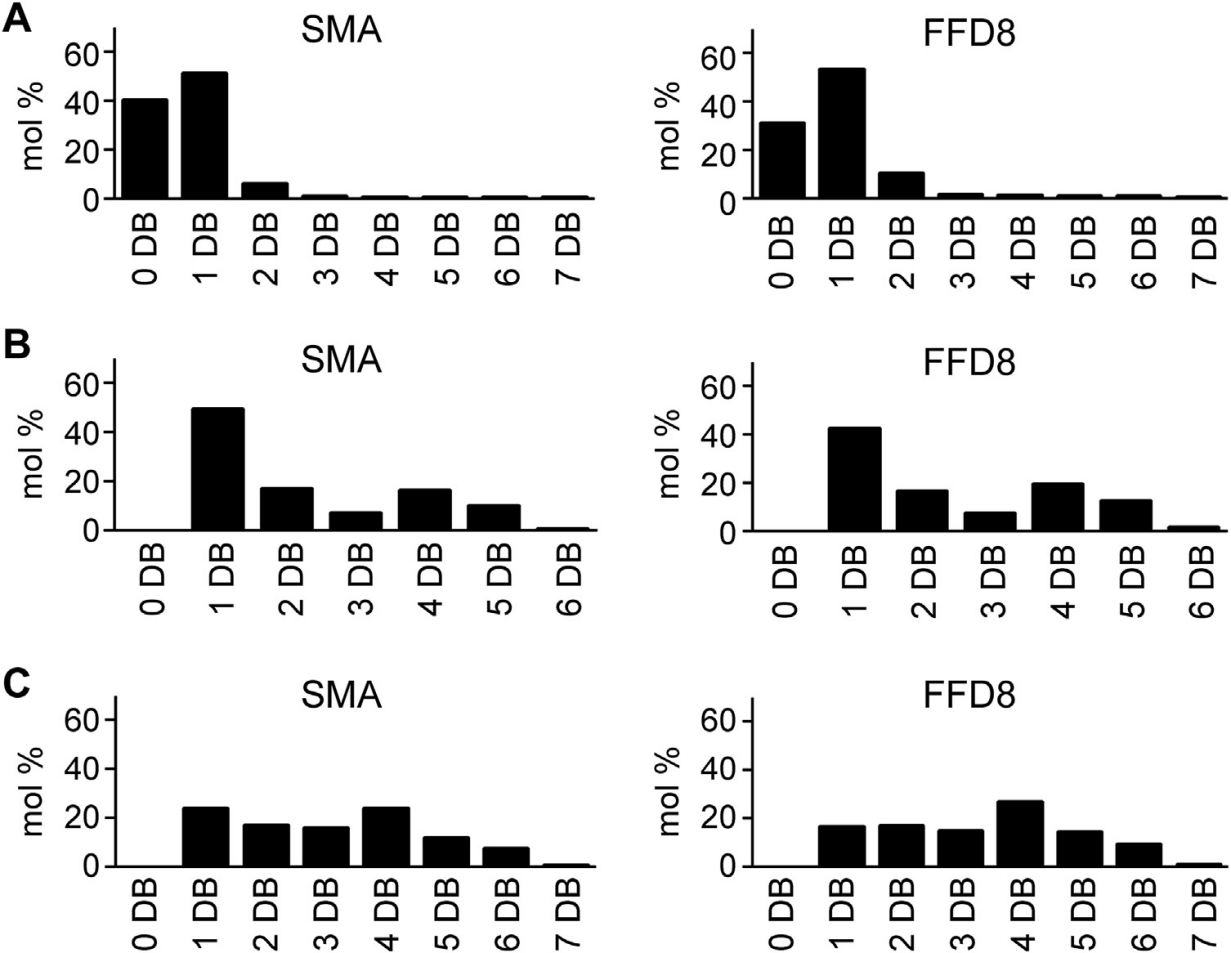
**More detailed comparison of the lipid composition of membrane fragments resistant to SMA *versus* FFD8.** Molar percentage of (*A*) total PC species based on the number (from 0 to 7) of double bonds (DB), (*B*) total PE species based on number (from 0 to 6) of double bonds (DB), and (*C*) total PI species based on number (from 0 to 7) of double bonds (DB) across fractions. Abbreviations as in Fig. 6. SMA, styrene-maleic acid.

As the FFD8 peptide (similar to SMA) apparently did not disintegrate the membrane raft microdomains, we compared the lipid composition of the membrane fragments resistant to solubilization by SMA or FFD8. These materials were obtained by sucrose gradient ultracentrifugation of the Jurkat membrane lysates followed by centrifugal concentration of the top fractions (see Experimental procedures below). As demonstrated in Figures 6 and 7, the lipid composition of the materials originating from the FFD8-treated membranes was similar to that of SMA-treated membranes and corresponded to the results of our previous study (15); both were characterized by high cholesterol content and predominance of lipids with saturated fatty acid residues.

### Cell membrane disintegration by other peptides

Based on the positive results with the FFD8 peptide, we purchased the following peptides (length similar to FFD8) and tested them for Jurkat cell membrane disintegrating capacity:

(a) Peptides incorporating F and D in other ratios or arrangements: FFFD6, FFFDD5, FDD8, FGFGDG4,
(b) Peptides containing other hydrophobic amino acid residues instead of one or both phenylalanines: FLD8, FID8, FVD8, FYD8, IID8, LLD8, VVD8.

**Figure 8:**
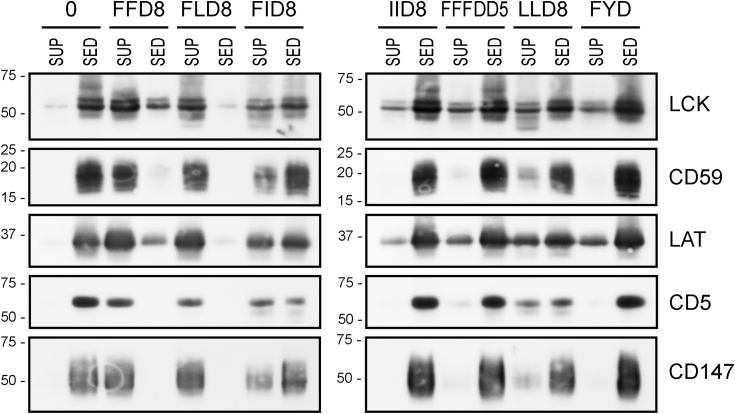
**Solubilization of Jurkat cell membranes by 1% solutions of the indicated peptides.** Five typical membrane proteins (LCK, CD59, LAT, CD5, CD147) were detected by immunostaining in the sediments and supernatants of the peptide-treated membrane samples. LCK, CD59, and LAT are characteristic membrane raft components, while CD5 and CD147 are nonraft proteins. Only the relevant parts of the blots are shown, corresponding to the area around the MW of the respective proteins. Positions of relevant m. w. standards (in kDa) are shown at the *left*. SED, sediment; SUP, supernatant.

As shown in Figure 8, only 1% solution of the FLD8 peptide solubilized membranes essentially completely similar to FFD8, while others (FID8, LLD8, IID8, FFFDD5, FYD8) did so only marginally, and yet others (FFFD5, FDD8, FVD8, VVD8, FGFGDG4, LGLGDG4) apparently not at all (not shown).

**Figure 9:**
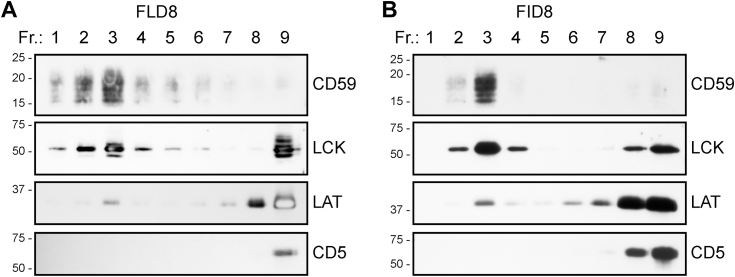
**Distribution of the indicated Jurkat cell membrane proteins in the density gradient ultracentrifugation fractions after membrane solubilization by the FLD8 and FID8 peptides.** Jurkat cell membranes were solubilized by 1% FLD8 (*A*) or 1% FID8 (*B*), the lysates were fractionated by density gradient ultracentrifugation, and the indicated proteins in the fractions were detected by immunoblotting. Positions of relevant m. w. standards (in kDa) are shown on the *left*. For details, see legend to Fig. 2*A*.

We also tested the possible effect of increased temperature (37 °C) on the solubilization power of some of the peptides disintegrating the membranes only partially. At 37 °C, the results with the FFD5, FFD7, IID8, and FYD8 peptides were very similar to those under room temperature (not shown). We also tested the separation by gradient ultracentrifugation of selected membrane proteins solubilized with two “non-FFD” peptides, namely FLD8 and FID8. As shown in Figure 9, the results were similar to those obtained with FFD8 (see Fig. 2 above).

**Figure 10:**
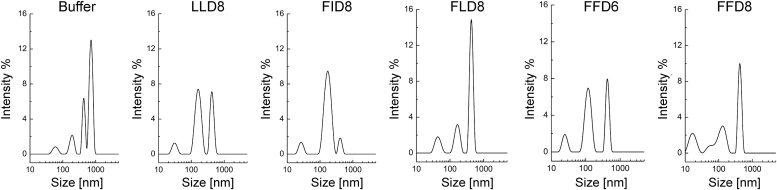
**MADLS analysis of the membrane lysates obtained by disintegration by the indicated peptides.** Jurkat cell membranes were solubilized by the 1% solutions of the indicated peptides and MADLS curves were recorded as described in paragraph 2.6. The negative control (“Buffer”) represents the sample handled without the addition of any peptide.

To characterize the size and heterogeneity of fragments resulting from cell membrane disintegration by the selected peptides, we performed the multi-angle dynamic light scattering (MADLS) analysis of Jurkat membrane lysates. As shown in Figure 10, the general pattern was similar in all cases—relatively large membrane fragments (around 400 nm, presumably derived from the membrane rafts) were present together with smaller ones. Only the sample solubilized with FFD8 contained the smallest particles (ca 15 nm, presumably corresponding to the nanodiscs/nanoparticles incorporating most of the membrane proteins). The sizes of the smallest particles in the samples solubilized by the other peptides (FFD6, FID8, FLD8, LLD8) were larger (ca 25–45 nm). These peptides apparently disintegrated the membranes into differently sized fragments, probably preserving more or less the native membrane nanodomains. In future studies, it would be interesting to characterize the nature of the “medium sized” entities (ca 100 nm).

It should be noted, though, that superficial inspection of the MADLS curves would provide a misleading impression about the relative amounts of the large *versus* small membrane fragments, as the signal intensity depends on the sixth power of the particle size and therefore is very strongly biased towards the larger species. Actually, the vast majority of membrane proteins are obviously present in the relatively small membrane fragments obtained by the peptide (or SMA) solubilization, as compared to the large PRMs (presumably membrane raft derived), as evidenced by the results shown in Figures 2*B* and 3*B*.

## Discussion

Hereby, we demonstrate that simple, homogeneous peptides structurally similar to the SMA copolymer disintegrate cell membranes in a similar manner to the copolymer. As expected, the peptides structurally most similar to SMA (general formula FFDn) were optimal for membrane solubilization. The basic repeated unit is the tripeptide FFD, in which two phenylalanine residues are chemically similar to the two hydrophobic styrene units in the copolymer, while the hydrophilic aspartic acid mimics one of the carboxyls of the maleic acid. The membrane solubilization power appears to depend on the peptide length, FFD7 being the necessary minimum. Interestingly, other possible peptide mimetics of SMA, such as FFFD5, FFDD6, or FFFDD5, solubilized the cell membranes only poorly or not at all. Another possible candidate, FFE7, in which glutamic acid replaced the aspartic acid was poorly soluble (not shown), probably because of the higher hydrophobicity of the glutamic acid side chain. Of course, it is possible that the performance of some of the nonsolubilizing peptide types could be improved if their length is increased, which has not been tested here. In any case, it seems that the peptides of the type FFDn (n > 7) and FXDn (X being L) are optimal and can be used as much more homogeneous analogs of the currently used nanodisc-forming copolymers.

With respect to the interactions with the membrane raft microdomains, the representative FFD8 peptide produced results similar to those observed in our previous study using the SMA copolymer (7). Thus, density gradient ultracentrifugation, gel filtration, and TEM (Figure 2, Figure 3 and 8) indicated that membrane raft microdomains containing LCK and CD59 were also resistant to disintegration by the peptide. Here again, as in our previous study using SMA (7), another raft protein LAT behaved differently, indicating that it probably had a different membrane lipid environment.

Following membrane disintegration by FFD8 (or SMA), the great majority of the membrane proteins are present in relatively small membrane fragments, somewhat incorrectly generally called ”nanodiscs” (see the critique of this term by Kamilar *et al*. (17)). It seems likely that the size of these entities is dependent on the copolymer or peptide used. Indeed, the results of the MADLS experiment (Fig. 7) indicate that the size of these smallest detectable complexes is about 15 nm in the case of FFD8 but approx. 40 nm in the case of FLD8. This might be potentially useful for future studies on close and more distant protein neighbors in the membranes.

Importantly, several of the tested membrane proteins could be readily immuno-isolated from the FFD8-solubilized membranes (Fig. 4), indicating that the presence of the peptide does not interfere with the interactions of the specific antibodies with their target antigens. Interestingly, in our previous studies, we observed that in the presence of SMA, CD5 could not be immunoprecipitated under otherwise the same conditions (unpublished observation).

An ultimate goal of the studies making use of the native nanodiscs obtained by membrane disintegration by suitable synthetic copolymers or peptides would be proteomic and lipidomic analysis following their specific immunoisolation. This should enable to follow, for example, changes in the close lipid environment of functionally important membrane receptors dependent on their activation or induced association with other proteins. This could not have been done in the present study because we used only a simple method of immunoisolation in which the immuno-isolated protein is released from the immunosorbent by drastic denaturating conditions. We are currently trying to optimize methods for elution under mild, non-denaturing conditions, minimizing nonspecific contamination.

The chemical nature of the peptides offers opportunities for useful straightforward modifications. In our recent experiments, we found that the FFD8 peptide C-terminally modified with FLAG tag (DYKDDDDK) or N-terminally with biotin also disintegrated Jurkat cell membranes (unpublished results). It should be noted that a recent paper reports membrane-disintegrating properties of another type of peptides (18). Last, it may be speculated that FFDn-like peptides encoded in an expression vector with specific (e.g. cancer) cell tropism might specifically kill such cells.

## Experimental procedures

### Reagents, cells, and antibodies

The peptides were custom-made by Apigenex and by Vidia (Vestec, Czechia). All the peptides were of a similar type, consisting of repeats of basic units, such as, for example, Phe-Phe-Asp (in short FFD). For the sake of brevity, their names here are based on the amino acid sequence of the basic unit followed by a numeral, indicating the number of repeats of the basic unit (e.g., FFD8 represents a peptide composed of an 8-times repeated FFD sequence). Human T cell line Jurkat was from ATCC; rabbit polyclonal antibodies to human LAT and Lck were kindly provided by Dr. L. Samelson and Dr. A. Veillette, respectively. mAbs to human CD5 (MEM-32), CD18 (MEM-48), CD147 (MEM-M6/1), LAT (LAT-01), LCK (LCK-01), and rabbit polyclonal antibody to human CD59 were prepared previously in our laboratory at IMG and are commercially available from EXBIO (Vestec). Biotinylated mAb L17F12 (CD5) and FITC-conjugated mAb MEM-48 (CD18) used in immunostaining of some western blots were from EXBIO, streptavidin-peroxidase conjugate from Thermo Fisher Scientific, peroxidase-conjugated anti-FITC mAb 5D6.2 from Sigma, goat anti-rabbit Ig-peroxidase and goat anti-mouse Ig-peroxidase from Bio-Rad.

### Cell membrane preparation (19)

Cells (1.5 × 10^8^) were suspended in 1 ml ice-cold hypotonic buffer (10 mM Hepes pH 7.4, containing 2 mM KCl, 5 mM MgCl_2_, protease inhibitor cocktail set III (Calbiochem)), incubated on ice for 15 min, and then passed 8× through the 25- and then 5× 30-gauge needle. The suspension was centrifuged 5 min at 580*g* and 2 °C to remove nuclei. The post-nuclear supernatant was centrifuged 10 min at 25,000*g* and 2 °C to pellet the membranes.

### Membrane and cell solubilization, SDS-PAGE, and Western blotting

The membranes prepared as above were lysed in 1 ml lysis buffer (20 mM Tris–HCl pH 8.2, containing 100 mM NaCl, 5 mM iodoacetamide, protease inhibitor cocktail set III (Calbiochem), 10 mM EDTA, 50 mM NaF, 10 mM Na_4_P_2_O_7_) without (negative control) or with 1% peptide. After 2 h at room temperature, the lysates were spun at 25,000*g* for 3 min to separate the insoluble materials from the solubilized ones. The sediments and supernatants were used for analysis by SDS-PAGE (nonreduced samples) and immunoblotting, selected supernatants also for gel filtration, immunoprecipitation, or gradient ultracentrifugation. In order to avoid problems with the presence of excess mouse immunoglobulins in the samples obtained by immunoprecipitation and analyzed by Western blotting, we used in some cases primary antibodies of rabbit origin (detection of LCK and CD59) or mAbs conjugated with biotin (CD5) or FITC (CD18), followed by appropriate peroxidase-conjugated secondary mAbs (see *2.1.*). To follow the membrane solubilization, we used Western blotting and specific immunostaining of several relatively abundant, immunologically relevant non-raft (CD5 (20), CD18 (21), CD147 (22)) and membrane raft–associated proteins (LCK (23), CD59 (24), LAT (25)). We found using this Western blotting–based approach more practical because the alternative, simple total protein staining in the fractions was complicated by the relatively high level of solubilized proteins (probably only loosely associated with membranes) even in the negative controls (not shown). The choice of specific membrane protein markers (nonraft or membrane raft–associated) was based mainly on the availability of suitable mAbs, mostly previously produced in our laboratory (and commercially available), the specificity of which has been confirmed in the HLDA Workshops.

### Density gradient ultracentrifugation

This method was performed as described before (26). Briefly, the membrane lysate (0.5 ml) was added to 0.5 ml of 80% (wt/vol) sucrose in lysis buffer and placed at the bottom of a 5.2 ml centrifuge tube, then carefully overlaid with 1.8 ml of 30%, 0.8 ml 20%, 0.8 ml 10%, and 0.7 ml 5% sucrose in lysis buffer and finally with 0.1 ml of lysis buffer. Centrifugation was performed at 10 °C in Beckman Optima MAX-E ultracentrifuge, using the MLS 50 swing-out rotor (18 h, 50,000 rpm). Nine 0.57 ml fractions were collected gradually from the top of the gradient; proteins were separated by SDS-PAGE and analyzed by immunoblotting.

### Sedimentation of disintegration-resistant membrane fractions by ultracentrifugation

Since the lipid concentrations in the top fractions of the density gradient were too low for lipidomic analysis, the combined relevant gradient fractions (1, 2, 3, 4, 5) were subjected to ultracentrifugation at 55,000 rpm (approx. 114,000*g*) for 1 h at 18 °C using a TLA 110 rotor in a Beckman Optima MAX-E ultracentrifuge. The sediments were used for lipidomic analysis.

### Gel filtration (size-exclusion chromatography)

Briefly, 0.1 ml membrane lysate was applied on top of a 1 ml Sepharose 4B column (in lysis buffer containing 1% peptide) and washed with the lysis buffer. The 0.1 ml fractions were collected (performed at room temperature) and analyzed by SDS-PAGE/immunoblotting. The initial fractions from this highly porous gel (fr. 4-6) contained large complexes or particles; the molecular weight standards IgM (900 kDa) and IgG (150 kDa) eluted mostly in fractions 7 and 8-9, respectively, while large particles (e.g. erythrocytes) eluted in void volume fraction No. 4. Selected membrane proteins in the fractions were detected by SDS PAGE/immunoblotting.

### Immunoprecipitation

Sixty microliters Protein A/G PLUS-Sepharose (Santa Cruz Biotechnology) bead suspensions were incubated with 5 to 10 μg of Abs in PBS for 2 h at 4 °C and washed with the lysis buffer. The antibody-coated beads were rotated for 2 h at room temperature with 250 μl lysates of the Jurkat cell membranes solubilized by the FFD8 peptide and washed on spin-columns (Bio-Rad) with lysis buffer. Immuno-isolated materials were eluted with 2× concentrated nonreducing SDS-PAGE sample buffer and analyzed by immunoblotting.

### Electron microscopy

Five microliters of each examined density gradient fraction were applied onto the glow-discharge–activated formvar/carbon-coated 300 mesh grid and let adsorb for 30 s. After that, almost all the drop volume on the grids was blotted off with a filter paper wedge, and the grids were negatively stained for 30 s with 2% uranyl acetate in double distilled water. Finally, the grids were blotted and air-dried at room temperature. The samples were examined on a Jeol JEM-1400 FLASH Transmission Electron Microscope (JEOL Ltd) at 80 kV. Digital images were recorded using a FLASH 2kx2k CMOS camera and saved in 16 bit tiffs. The recorded images were processed in the AnalySis5.2 software (www.emsis.eu; formerly Olympus Soft Imaging Solutions) suite (EMSIS GmbH) using an embedded module (Optimize 16-bit image for 8-bit display). No other image manipulation was used.

### Lipidomics analysis

#### Sample preparation

A volume of 180 μl of cold methanol containing a mixture of lipid internal standards (CL 16:0/16:0/16:0/16:0, 332.6 pmol; Cer 18:1;2O/17:0, 32.6 pmol; cholesterol(*d*_7_), 457.6 pmol; DG 18:1/18:1(*d*_5_), 28.8 pmol; HexCer 18:1;2O/17:0, 25.2 pmol; FA 18:1(*d*_9_), 61.8 pmol; LPC 17:1, 71.0 pmol; LPE 17:1, 96.7 pmol; PC 15:0/18:1(*d*_7_), 23.9 pmol; PE 17:0/17:0, 100.1 pmol; PG 17:0/17:0, 116.5 pmol; PI 15:0/18:1(*d*_7_), 21.3 pmol; PS 17:0/17:0, 1013.7 pmol; SM d18:1/17:0, 50.2 pmol; TG 17:0/17:1/17:0(*d*_5_), 21.1 pmol) was added to each sample containing sediments, followed by shaking (30 s) and sonication (3 pulses). Then, 600 μl cold methyl tert-butyl ether was added, followed by shaking (30 s), addition of 175 μl water, shaking (30 s), and centrifugation (16,000 rpm, 10 min, 4 °C). A volume of 300 μl of the upper organic phase was collected and evaporated. Dried lipid extracts were resuspended in 100 μl methanol containing the internal standard 12-[[(cyclohexylamino)carbonyl]amino]-dodecanoic acid (200 ng/ml). After shaking (30 s) and centrifugation (16,000 rpm, 5 min, 4 °C), the extracts were submitted to LC-MS analysis.

#### LC-MS analysis

The LC-MS systems consisted of a Vanquish UHPLC system (Thermo Fisher Scientific), an OptaMax NG ion source with a HESI-II probe (Thermo Fisher Scientific), and an Orbitrap Exploris 480 mass spectrometer (Thermo Fisher Scientific). Details of LC-MS methods used for lipidomic profiling can be found elsewhere (27, 28).

#### Data processing

MS-DIAL (v. 4.9.221218) (http://prime.psc.riken.jp/compms/msdial/main.html) software program (29) was used for data processing. Lipids were annotated using accurate mass and MS/MS matching with the LipidBlast library in MS-DIAL. Over 670 unique lipid species covering 22 lipid classes were annotated (Table S1). Quantification was performed using class-specific internal standards except for GM3 and Hex2Cer species, in which internal standard HexCer 18:1;2O/17:0 was used. Results were expressed in pmol of particular lipid species per isolated membrane fraction followed by calculating relative molar concentrations of particular lipid classes (%).

### Multi-angle dynamic light scattering

MADLS measurements were carried out on Zetasizer Ultra ZS (Malvern Panalytical Ltd). A 633 nm He–Ne laser light source and an avalanche photodiode detector were used. The scattering intensity was collected at the temperature of 22 °C at angles of 13°, 90°, and 173° and data were analyzed using the ZS XPLORER software (v3.22) (https://zs-xplorer.software.informer.com/3.2/).

## Data availability

Representative experiments are shown in the figures. For any additional information, please contact the corresponding author.

## Supporting information

This article contains supporting information.

## Conflicts of interest

The authors declare that they have no conflicts of interest with the contents of this article.
